# Pathology and Practice *(Medical)*

**Published:** 1818-03

**Authors:** 


### PART III.

### MONTHLY SKETCH OF FOREIGN MEDICAL SCIENCE.

——— Sparsa Colligens.

### I. PATHOLOGY AND PRACTICE *(Medical).*

As the Foreign Journals, now before us, contain nothing of importance relative to *Anatomy* or *Physiology*, we embrace this opportunity of laying before our readers a few extracts, some time since prepared, from a work which professes, in no ordinary tone of confidence and presumption, to throw new light upon diseases.* The production of M. Prost, although it at first attracted some notice on the continent, appears scarcely to be known, even by name, among the practitioners of this country. Our usual method of arrangement will be followed in the cases which we are about to cite.

1. *Thoracic Diseases.* In our last Sketch † was recorded a fatal case of laryngeal inflammation. This affection, although commonly of acute character and rapid progress, we may here take occasion to remark, sometimes commences in an indolent and insidious manner, or, when at first active, assumes this form under the influence of feeble and inefficient treatment. Ulceration ensues; and thus a disease is formed, closely resembling, in its general physiognomy, pulmonary consumption, and usually indeed by the *trading* practitioner confounded with it. Of this affection, first denominated *laryngeal phthisis* by the French, the following is a good example:

COGNARD, aged 33, of a thin habit, had enjoyed good health till within a year past. One day, when much heated, he sat down

* *Médecine eclairée par l'Observation et l'Ouverture des Corps. Par P. A. Prost,* &c. 2 tomes, octavo, Paris.

† *Medico-Chir. Journal,* vol. v. page 152.

in a current of air, by which he was chilled. Shortly afterwards, he felt a pain stretching from the throat to the breast, accompanied by cough. The complaint in his throat got worse, and for which he was received into the hospital at Strasburgh, but without experiencing any benefit there. In a short time, he came to Paris, where his voice was so affected that he could scarcely make himself understood. The pain in the throat was constant, and accompanied by a sense of coldness in the part, fatiguing cough, saltish mucous expectoration, and bad taste in the mouth. A heavy dull pain was felt in the chest; there were night sweats; mid-day and evening accessions of fever; progressive emaciation. When received into La Charité (March 1st, 1803), the patient presented the following appearances: Countenance wan; cheeks flushed in the centre; respiration short, and suffocative; cough constant; voice so feeble as scarcely to be heard; expectoration purulent; sense of pruritus in the throat; deglutition difficult and painful; perspiration in the night colliquative; febrile heat of skin; pulse small and frequent; febrile exacerbations, as before mentioned. From this time, for five or six weeks, his symptoms got worse and worse. Aphonia was now complete. He was harassed with hiccup; his belly swelled; and, for a few days previous to dissolution, some inflammatory symptoms were developed.

*Dissection.* The brain was sound, excepting some thickening of its mucous membrane; the pharynx was unaffected. There was an ulceration of the superior and middle part of the larynx, comprehending the posterior half of the glottis. The left aretenyoid cartilage was partly ossified and carious. The surface of the cricoid cartilage was covered with red purple-like elevations. The membrane covering the right arytenoid cartilage was thickened and ulcerated. The superior ligaments of the epiglottis were thickened and fretted (ronges): a slight ulceration at the base of this cartilage. The ventricles of the larynx were little affected interiorly; but the chordæ vocales were much thickened, and the left one ulcerated. The thyroid cartilage was sound; the posterior part of the cricoid ossified. The mucous membrane of the larynx, generally, thickened, and as it passed down the trachea presenting some red spots. The lungs were partially adherent; and some small greyish tubercles were found on and near their surface. The bronchial glands were large and firm; heart natural. In the abdomen some ulcerations were found on the inner surface of the intestines; the mesenteric glands were indurated and enlarged; and about a quart of serum in this cavity.

Mr. Hodgson, in his valuable work on Aneurism,* observes, that aneurism of the thoracic aorta may give rise to a train of symp-

---

* *Treatise on the Diseases of the Arteries and Veins.* page 93.—See *Morbid Anatomy of the Liver,* or our Review of it, *Med. Chir. Journal,* vol. i.

toms very difficultly distinguished from those of pulmonary phthisis; and that, in such cases, the character of the expectoration constitutes the most striking, if not the only diagnostic sign. Complication of phthisis with aneurism is, we apprehend, a rare occurrence. That, however, it may occasionally exist, the ensuing history sufficiently evinces.

Bathe, a painter, of a bilious temperament, experienced, in the 45th year of his age, a cough, which frequently returned afterwards with glairy and mucous expectoration. About this time, he also began to complain of pain in the left hypochondrium, which, increasing with the cough, became very distressing, and appeared to shoot along in the direction of the vertebral column. In this state he continued during twelve months, when, it being the winter-time, the expectoration became thicker; the pain in the hypochondrium more acute, and extended; emaciation increased: there was sleepiness; increased pain on bending the trunk; pain about the region of the heart. The symptoms characteristic of pulmonary phthisis now supervened: to these were once added suppression of urine. All these symptoms getting gradually worse, the patient was carried off.

*Dissection.* The vessels of the brain and its membranes were somewhat engorged; about five drachms of water in the lateral ventricles. Tubercles in a state of suppuration, and communicating freely with various branches of the bronchial tubes, were pretty numerous in the right lung, in which were also found two cysts, one the size of a pullet's egg, partly cartilaginous, partly ossified. The left lung was less diseased, and a small, hard, round body was found in it. Heart natural. In the aorta, from its origin, were seen scales of ossification formed in the tissue of the inner coats. In the thoracic portion of this vessel, not far from the diaphragm, were two small aneurisms.

In the abdomen there was nothing remarkable, except a considerable ulcer occupying the inner and middle coats of the cæcum, and a similar one in the colon.

In an interesting case of *Cardi-pericardic Adhesion, complicated with Ossification and Hydrothorax*, Dr. Prost does not specify the existence of that pulsation *in epigastrio*, which Burns considers so characteristic of the adhesion of the pericardium to the heart.

Usse, ætat. 63, an attendant on the sick, had enjoyed but a valetudinary state of health for several years. An habitual dyspnœa often obliged him to give up his employments. He could not sleep in bed, without having his head and shoulders much elevated; he became progressively debilitated. On the 12th of November, he found himself more than usually fatigued; 15th, took to his bed; his breathing much oppressed; frequently changed colour; felt cold chills creep over his body. Nevertheless, his appetite continued keen, and he did not emaciate. For a long time, the heart beat over a large space, and irregularly. No swelling of the extremities. These symptoms continued, without any material alteration for some months, when fever developed itself, accompanied by daily exacerbations, in which delirium was manifested. Symptoms of debility next presented themselves; constant loquacity; urine free; belly costive. Two days before death, his skin became covered with pimples and red spots. Still the symptoms were partly inflammatory, but accompanied with great prostration of strength. Death.

*Dissection.* In the *head*, there was some serous effusion between the membranes. Substance of the brain soft and watery, two drachms of water in each ventricle; half an ounce at the basis cranii. *Thorax.* The left lung was partially adherent, somewhat gorged, but crepitous. The cavity of the pleura on this side contained a pint of deep red, sanguineous-looking serum. Right lung universally adherent, but crepitous throughout. The pericardium was completely adherent to, and identified with, the surface of the heart. This adhesion appeared of very remote formation. At the lower part was an osseous lamina of very irregular figure, about two inches in diameter, taking the form of the heart at that place, and into the substance of which, several osseous prominences dipped. This ossification was, in one place, more than four lines in thickness. The heart itself was considerably enlarged, its cavities filled with black blood, and many specks of ossification on the different valves, particularly of the left ventricle. Liver and spleen greatly enlarged.

2. *Abdominal Diseases.* There are few experienced and candid practitioners, who will not confess that they have repeatedly witnessed the failure of mercury and every other remedy, however judiciously employed, in certain forms of hepatic disease. Dr. Farre, whose authority on this point we are by no means disposed to question, even asserts, that, in all those varieties of tumour of the liver, to which he has applied the term *tuber*, mercury, liberally employed, constantly aggravates the disease, and hastens its progress. We have, ourselves, more than once noted the inutility, if not the baneful effects, of this remedy in that tuberculated state of the hepatic organ, of which the ensuing case exhibits a good illustration. The treatment here is not specified.

Rousseau, ætat. 59, of a sanguineous temperament, and inclined to obesity, had been long subject to bilious attacks, which generally gave way, for a time, to emetics. On the 10th of November, he was affected with indigestion, vomiting, and diarrhœa. On the 13th he was seized with a sharp pain in the abdomen, accompanied by impeded respiration, but without any distinct pain in the thorax. His hands swelled; the belly became more prominent; the pain and dyspnœa now disappeared; and a diarrhœa alone remained: he found his strength diminish. On the 27th, the bowel complaint still continued undiminished by any remedies, and he now perceived that his skin had acquired a yellow tinge: no pain in the abdomen.—On the 29th, he was admitted into La Charité, presenting the following symptoms; viz. Face and eyes yellow; abdomen swelled, with fluctuation, but no pain. All the extremities, particularly the lower, œdematous; tongue dry and whitish; thirst ardent; lips blackish; anorexy; prostration of strength; harassing diarrhœa; restlessness; skin cool; pulse small, weak, and somewhat accelerated.—December the 1st. No alteration. His drink vomited since last report; can lie on either side; prostration of strength still greater. The diarrhœa and other symptoms continued to harass the patient till the 4th, when he expired.

*Dissection.* General anarsarca. Some infiltration between the coverings of the brain, which was soft, and presented no red specks when cut into. The ventricles considerably distended with serum, four ounces at the basis cranii.—*Thorax.* Lungs free and crepitous. Some serous fluid, however, could be squeezed from their parenchymatous structure. No water in the sacs of the pleura. Heart rather large and covered with fat. Aorta descendens very small in calibre: where it passes under the diaphragm, the vessel was wrinkled and ossified.—*Abdomen* contained about a pinte [French] of serum. Omentum and mesocolon very fat. Liver of an extraordinary size, of a bronze colour, but free from adhesions. From the various sections into the substance of this organ flowed a liquid, albumino-purulent matter, of a yellow colour [une matière liquide, albumino-purulente et jaunatre]. This matter was contained in cysts of a very small size, and very close to each other; they varied in magnitude from that of a large pea to a small pin's head. The intermediate texture of the liver was red, very soft, and easy of laceration. The gall-bladder little prominent, and containing little bile, but a large triangular gall-stone, which seemed to obstruct the ductus cysticus. Pancreas cartilaginously indurated.

Granting the case, which we shall next introduce, to have been *colica pictonum,* the post-mortem appearances and living phænomena displayed by it, must impress on the mind of the intelligent British practitioner, a melancholy but instructive lesson. What might not *venesectio ad deliquium,* the warm bath, fomentations, leeches, cupping, mercury, and purgatives, with or without opium, have done here. Surely, no one can peruse the narrative without experiencing the most lively emotions of interest, and without reaping advantage from the perusal.

Simon Masse, ætat. 32, a ship painter, of a sanguineo-lymphatic temperament, chesnut-coloured hair, and pale complexion, had always enjoyed good health; and during the last five years that he had been employed in working among paints, and grinding the oxyds of lead, copper, arsenic, &c. he suffered no indisposition. One night, having supped pretty heartily, he was seized with a most violent colic, succeeded by vomiting, first of those substances on which he had supped; afterwards, of yellow and bitter matters. —*2d day.* After taking some warm wine he again vomited. Had no stool for several days. He was now admitted into La Charité, with the following symptoms:—Frequent hiccupings; vomiting; violent colicky pains; constant watching. Two glysters procured the evacuation of some hardened fæces.—*3d day.* Skin slightly tinged yellow; tongue moist and white; *pulse full, hard, and frequent;* the hiccup augmenting the colicky pains; abdomen tense, swelled, and very painful, especially about the umbilicus, *where the slightest pressure caused exquisite pain. A gentle emetic*, which brought off much bilious and bitter stuff from the stomach. The night more tranquil than the preceding: sudorifics, purging glysters, and an anodyne.—*4th day.* Vomiting of similar matters as yesterday; abdominal pain irritated by the singultus. At mid-day, a cessation of the vomiting, and some sleep.—*5th day.* The eyes inflamed; tongue dark coloured; thirst; belly tense, swelled, and painful on pressure; cannot lie on either side; lies on his back; constipation obstinate; glysters returned unaltered; very little sleep; pulse full, hard, but less frequent. Same medicines as on the third day.—*6th day.* Cheeks flushed; little thirst; hiccup less frequent; abdomen as yesterday. No evacuations, notwithstanding the glysters; little or no sleep; pulse hard, frequent, and irregular. Patient not anxious about the issue of his disease.—*7th day.* Countenance highly flushed. Some appearance of perspiration on the skin this morning. No sleep; jactation; belly extremely painful on pressure; pulse softer; Constipation obstinate. *Eau de Casse, tisane sudor.*—*8th day.* An *emetic* brought off abundance of yellow and bitter matters; appeared better afterwards. Patient has hopes of recovery; has had frequent stools during the day. Same remedies.—*9th. day.* The patient experienced a sudden change this morning, with great prostration of strength. The face soon afterwards turned livid; the pulse small, weak, and irregular; convulsions; death.

*Dissection.* No emaciation. Countenance still expressive of pain. *Head.* The sinuses and vessels of the meninges gorged with blood. Brain firm, about two drachms of water in each ventricle. Thoracic viscera sound.—*Abdomen.* In the cavity of the peritonæum, a pinte [French] of yellowish milky serum, like very thin pus. The membrane itself injected, and of a red, violet colour in various parts. Mesocolon of a red brown colour, and highly vascular. Between the circumvolutions of the intestines a purulent-looking liquid. No adhesions of the intestines. A reddish circle about the cardia. The internal coats of the small intestines presented nothing remarkable. The peritonæal tunic was highly vascular, and the vessels gorged with black blood. Ilium contained a fluid-like thin pus. About six inches from the cæcum, the ilium began to assume a livid aspect, and M. Bayle hardly touched it with the forceps, when the gut fell in pieces. This disorganization appeared confined to the peritonæal and muscular coats of the bowel, the mucous membrane being very little altered. The colon and rectum were extremely contracted; their internal membrane thin, and formed into numerous folds resembling the sigmoid valves of the arteries. Small quantities of a yellowish, almost solid matter, were found in these parts of the canal, jammed, as it were, by the above-mentioned valvular folds. The peritonæal coverings of these large intestines were equally vascular, and gorged with blood, as those of the small. The liver of a bronze colour externally, but natural within. The gall-bladder distended with green bile. No other remarkable appearance.

3. *Febrile Diseases.* The recital of a case of *inflammatory fever with violent determinations to various organs*, will, from the utter neglect of all vigorous treatment, fully justify the observations which we have occasionally made on the inert and temporizing character of French practice in this numerous class of maladies, among which the triumphs of well-directed art are much more frequently and strikingly exhibited, than in affections of a chronic nature.

Thibault, a locksmith, 51 years of age, of a sanguineo-bilious temperament and athletic constitution, enjoyed habitual good health. From the 5th till the 11th of June, he had worked with extraordinary exertions at the forge till late every night. During this time, he had frequently exposed himself to the cool air when overheated with work. On the latter day (11th), he complained of sense of weariness; and all at once he was seized with a rigor, followed by an extreme prostration of strength,—(Courbature)*—violent headache, urgent thirst, and bitter taste in the mouth. Cough next ensued, and soon became obstinate, with difficulty of breathing and bloody expectoration. In the evening, great restlessness; and the night was passed without sleep. On the succeeding day he was received into La Charite, where he presented the following symptoms; viz. Countenance indicative of suffering; respiration difficult, laborious, and quick. Cough almost incessant; acute pain under the sternum on the right side. The slightest percussion on this spot caused the most terrible sufferings to the patient, who otherwise bore his illness with great fortitude: that side of the chest sounded obscurely. Skin dry and burning; tongue of a vermillion redness at the edges, and coated in the middle with yellow mucus; thirst urgent. The patient was constantly tossing about his arms and legs; eyes prominent; cheeks of a red violet colour and shining; could only lie on his back, with his head and shoulders elevated. *Pulse full, bounding, tensive, and quick.*

These symptoms became rapidly exasperated. In the evening, the eyes were starting from their sockets; respiration extremely difficult. The patient seemed almost suffocated in the act of inspiration; mouth wide open; nostrils dilated; eyes starting; delirium; death about midnight. It does not appear that any active

---

* *Courbature* (acerba lassitudo), "the name of a disease very common among persons subjected to laborious occupations. It is characterized by obtuse pains in the arms, legs, and back, by depression of strength, extreme lassitude, and general torpor of the animal machine."—*Dictionaire de Médicine.*

measures, if, indeed, any measures at all, were taken, to check the furious storm of this disease !

*Dissection.* External appearance. The corpse, muscular and fat; face, neck, and shoulders, of a violet-red colour.—*Head.* All the blood-vessels, external and internal, particularly those of the meninges of the brain, were gorged with black blood. Effusion of lymph between the membranes ; sinuses filled with black blood ; substance of the brain firm, and all its sections exhibiting numerous red points; water in the lateral ventricles. Mucous membranes of the pharynx and larynx inflamed.—*Thorax. Left* lung free from adhesions, crepitous, and sound. *Right* lung adhered to the sternal portion of corresponding pleura by an albuminous crust, easily lacerable. Substance of this lung gorged with a sanguineous fluid—pieces of this lung, when thrown into water, sank forthwith. The bronchia of a violet colour to their minutest ramifications. Costal pleura of this side inflamed, particularly near the point of adherence under the sternum. The heart rather high coloured. *The internal surfaces of the great vessels issuing from the heart lined with tubes of coagulable lymph, which required the scalpel to separate them from the surfaces of the corresponding internal tunics. Abdomen.* A red circle of inflammation round the cardiac orifice of the stomach. Intestines sound. Liver very much enlarged—in colour, of a dull and unequal red ; in substance, very soft. System of the vena portæ gorged with blood. In the gall-bladder, a little green bile. Spleen double its natural size.

This Case offers a memorable specimen of the ravages which an inflammatory fever can commit on the human frame, under the imbecile treatment pursued by our continental brethren. The most concentrated endemic fever—the plague itself, hardly ever cuts the thread of life with greater rapidity than in the foregoing instance. Can any one peruse this case without exulting in British practice? The youngest pupil on this side of the Channel would have recognized, from the first invasion of the disease, the disorganization which was impending, and he would have *checked*, at least, its march, if he did not entirely subdue it.

The details of a dissection of *this kind* are invaluable ; because they offer a faithful picture of the natural effects of acute diseases on the interior organs. *Here* the picture can seldom, if ever, be seen, since the feeblest practitioner counteracts, in some degree, the ravages of the malady. On the Continent, the FEVER FIEND is seen to pounce upon one organ after another, with the greatest *sung froid*, on the part of the physician, while the ptisan, the eau sucré, or the lavement, but feed the flame that is devouring the patient's vitals !

We shall bring forward one more Specimen from the ranks of *Fever:*—BENDER, a painter, ætat. 20, of a sanguineo-lymphatic temperament, and who had enjoyed good health in general, was seized, on the 14th of April, all at once with a strong rigor, which lasted from mid-day till the evening. The mouth became dry; thirst urgent; head-ache violent; skin burning; great nausea and inclination to vomit; general prostration of strength. Cough came on, and continued incessant. Blood soon appeared in the expectoration.

The night was passed without any sleep, and in great agitation. Burning flushes frequently pervaded the surface of the body, but were not followed by perspiration. These symptoms were all exasperated during the next two days, in which time there were no evacuations by stool, nor any transpiration through the skin. 17th. Cough most harassing; expectoration thick, and in colour resembling the dregs of wine. No alleviation of the symptoms. Constipation still continues; urine high-coloured and scanty; pulse full, expansive, and quick. In the night the agitation greater than ever; no sleep; tendency to delirium. 18th. The cheeks highly flushed; patient appears overwhelmed with sufferings; tongue dry and black; abdomen tender; respiration difficult, short, and quick. Cough less; expectoration more scanty; pain in the right side of the chest; bowels opened; in the evening an exacerbation. 19th. An alleviation of the symptoms; three stools. 20th. The remission still more considerable; but in the evening an exacerbation again took place; bowels open. 21st. A still greater exasperation of the symptoms. Head-ache violent; tongue dry, and brown; pulse small and frequent. 22d, 23d, and 24th, nearly the same. 25th. Symptoms of prostration of strength. 1st of April. The cheeks very red; mouth dry; thirst; pulse small, the pulsations running into each other; pains in the hypochondria. 2d. Dragging pain in the right breast, especially on moving; nausea and vomiting of yellow matters. At night a great exacerbation with constant delirium. 3d. The same. 4th. Inability to lie on the right side; right cheek very red. 5th. Has had no stool for four days; great pain in the right side; percussion there cannot be borne; sounds badly. 6th. The same. 7th. Death closed the scene.

*Dissection.* Body not emaciated; no œdema; abdomen tense and swelled; (Ballonné) face, neck, and shoulders of a violet colour. *Head.* All the vessels of the integuments, of the meninges, and of the brain were very much gorged with thick, black blood (fort gorgés d'un sang noir et epais.) The substance of the brain firm; great number of red points in the medullary substance throughout all its sections; about two drachms of serum in each ventricle. *Thorax.* Left lung sound and crepitous; pleura of that side unaltered; the bronchia a little reddened. The right lung firm, red, and gorged with sanguino-albuminous fluid, was in the first stage of carnification (dans le premier état de carnification). This lung adhered to the diaphragm by a thick layer of recently deposited coagulable lymph, easy of laceration; some elongated adhesions also to the costal pleura. The pleura of this side, pulmonary, costal, and diaphragmatic, highly inflamed. In this bag of the pleura were effused three half-pints (English) of reddish serum. [trois demi-setiers.] Two ounces of water in the pericardium. *Abdomen.* Spleen large, red, and soft; liver of a pale red colour; soft, and easily torn.

Such,— to conclude with the words of the able and eloquent writer by whom the materials of our present Sketch have been contributed,—Such is a genuine picture of the effects of the "*medecine expectante*" in acute diseases! Brunonianism itself is infinitely more charitable, inasmuch as nature is soon spurred on to destruction between the disease and the remedy; while here she retreats step by step, combating a dreadful malady, while the physician calmly looks on, and records the ravages of the disease and the struggles of nature, without attempting to check the one or assist the other.

We are under the necessity of deferring some Papers on *Animal and Vegetable Chemistry*, intended for the present, to our next Number. EDITORS.

---

